# Systematic review of quality of life following pleurectomy decortication and extrapleural pneumonectomy for malignant pleural mesothelioma

**DOI:** 10.1186/s12885-018-5064-4

**Published:** 2018-11-29

**Authors:** Rebecca M. Schwartz, Wil Lieberman-Cribbin, Andrea Wolf, Raja M. Flores, Emanuela Taioli

**Affiliations:** 1 Department of Occupational Medicine, Epidemiology and Prevention, Northwell Health Physician Partners, Hofstra Northwell School of Medicine, Great Neck, NY USA; 2 Institute for Translational Epidemiology and Department of Population Health Science and Policy, Icahn School of Medicine at Mount Sinai, New York, NY USA; 3 Department of Thoracic Surgery, Mount Sinai Health System, Icahn School of Medicine, New York, NY USA; 4 Tisch Cancer Institute, Icahn School of Medicine at Mount Sinai, One Gustave L. Levy Place, Box 1133, New York, NY USA

**Keywords:** Outcomes, Functional measures, Cancer, Surgical approach

## Abstract

**Background:**

Few studies have focused on quality of life (QoL) after treatment of malignant pleural mesothelioma (MPM). There are still questions as to which surgical procedure, extrapleural pneumonectomy (EPP) or pleurectomy decortication (P/D) is most effective and results in better survival outcomes, involves fewer complications, and results in better QoL. Here we performed a literature review on MPM patients to assess and compare QoL changes after P/D and EPP.

**Methods:**

Research articles concerning QoL after mesothelioma surgery were identified through May 2018 in Medline. For inclusion, studies were 1) cohort or randomized controlled trials (RCT) design, 2) included standardized QoL instruments, 3) reported QoL measurement after surgery, 4) described the type of surgery performed (EPP or P/D), 5) were written in English. Measures of lung function (FEV1, FVC) and measures from the EORTC-C30 were compared 6 months following surgery with preoperative values.

**Results:**

QoL data was extracted from 17 articles (14 datasets), encompassing 659 patients (102 EPP, 432 P/D); the available evidence was of low quality. While two studies directly compared QoL between the two surgical procedures, additional data was available from one arm of two RCTs, as the RCTs were not comparing EPP and P/D. The remaining data was reported from observational studies. While QoL was still compromised 6 months following surgery, from the limited and low quality data available it would appear that P/D patients had better QoL than EPP patients across all measures. Physical function, social function and global health were better at follow-up for P/D than for EPP, while other indicators such as pain and cough were similar. Forced Expiratory Volume (FEV1) and Forced Vital Capacity (FVC) were reported in one study only, and were higher at follow-up for P/D compared to EPP.

**Conclusions:**

Although the existing evidence is limited and of low quality, it suggests that P/D patients have better QoL than EPP patients following surgery. QoL outcomes should be factored into the choice of surgical procedure for MPM patients, and the possible effects on lung function and QoL should be discussed with patients when presenting surgical treatment options.

## Background

Malignant pleural mesothelioma (MPM) is a fatal malignancy that remains a global concern. The prominent risk factor for MPM is occupational or environmental exposure to asbestos [1–6]. It is estimated that MPM occurs in 2000 to 3000 people annually in the United States [1], while a study of 19,134 cases from the U.S. National Cancer Database from 2004 to 2013 reported the total number of mesothelioma cases in the U.S. ranged from 1800 to 2000 cases per year [7]. The global incidence of mesothelioma is predicted to increase; it was estimated that 9000 cases will occur Western Europe in 2018 [8], with peak incidence occurring in 2025 in Japan [9], leading to hundreds of thousands of deaths. Several population-based studies on MPM patients have reported a median survival of 8–11 months [10, 11], 8 months [12], 9.5 months [13], 9.2 months [13], and 10.7 months [13] under various treatments. Current treatment options include combinations of surgical resection, radiation, and chemotherapy, with chemotherapy administered as neoadjuvant and adjuvant therapy [1, 14–16]. A previous analysis of 14,228 MPM patients using the Surveillance, Epidemiology, and End Results (SEER) database reported that surgical treatment was an independent determinant of extended survival [17]. An additional SEER analysis of 5937 MPM patients from 1990 to 2004 reported that surgical treatment was performed in 22% of cases [18].

Although the role of surgery in MPM patients remains controversial [1, 7], extrapleural pneumonectomy (EPP) and pleurectomy (P/D) are the most common surgical procedures performed. Our group has previously shown that differences in survival between the two procedures are modest, but favor P/D in both short- and long-term survival [19]. A separate meta-analysis reported that EPP patients had significantly higher 30 day mortality compared to P/D patients as well as higher postoperative complications [20]. Given the mortality and morbidity associated with surgical resection for MPM, it is important to determine if there is a difference in QoL following EPP and P/D in order to inform patients and guide treatment choices. Here we build on previous work [21] and compare published QoL results for patients undergoing EPP or P/D for MPM at 6 months following surgery.

## Methods

### Search strategy and selection criteria

A literature search was performed in PubMed using the search terms “quality of life” AND “mesothelioma” AND “surgery” through May 2018. Three meta-analyses on mesothelioma surgical outcomes [22–24] and two reviews on QoL after mesothelioma treatment [21, 25] were searched for QoL data after surgical resection for MPM. The reference lists from articles retrieved from this database were also reviewed and evaluated for eligibility. No limit on the year of publication was imposed on the search; however inclusion criteria were the following: 1) cohort or randomized controlled trials design (RCT), 2) studies that used standardized QoL instruments, 3) studies reporting QoL measurement after surgery, 4) studies describing the type of surgery performed (EPP or P/D), 5) articles written in English.

### Data extraction

All relevant descriptive information was extracted from each study including author, year of publication, study design, years of data collection, number of patients included, case selection, tumor histology, type of surgery, other adjuvant cancer treatments, type of QoL questionnaire utilized, time intervals that QoL data was reported, and time to recurrence. Authors were not contacted for additional data. The primary outcome of this study was changes in QoL following EPP and P/D surgery. Data was extracted independently by three reviewers (ET, AW, WL-C). In cases of disagreement during data extraction, a final decision was made by a fourth reviewer (RS). The National Institutes of Health (NIH) Quality Assessment Tool for Observational Cohort and Cross-Sectional Studies in conjunction with extracted data from each study was used to determine the internal validity, risk of bias, and quality of included studies [26]. We followed the PRIMSA Checklist [27] in reporting our study.

### Statistical methods

The mean and standard deviation for each available QoL item were extracted from each study. The difference in mean baseline and mean follow-up scores from articles that reported QoL with the European Organization for Research and Treatment of Cancer (EORTC) QLQ-C30 were calculated. When cases were stratified by their Performance Status (PS) score, baseline QoL measures were weighted by the number of PS 0 and PS 1–2 patients to calculate an overall baseline score and the follow-up score was calculated from this baseline value. Due to the heterogeneity and the lack of comparable data across studies, additional pooled analyses could not be performed.

## Results

**Fig. 1:**
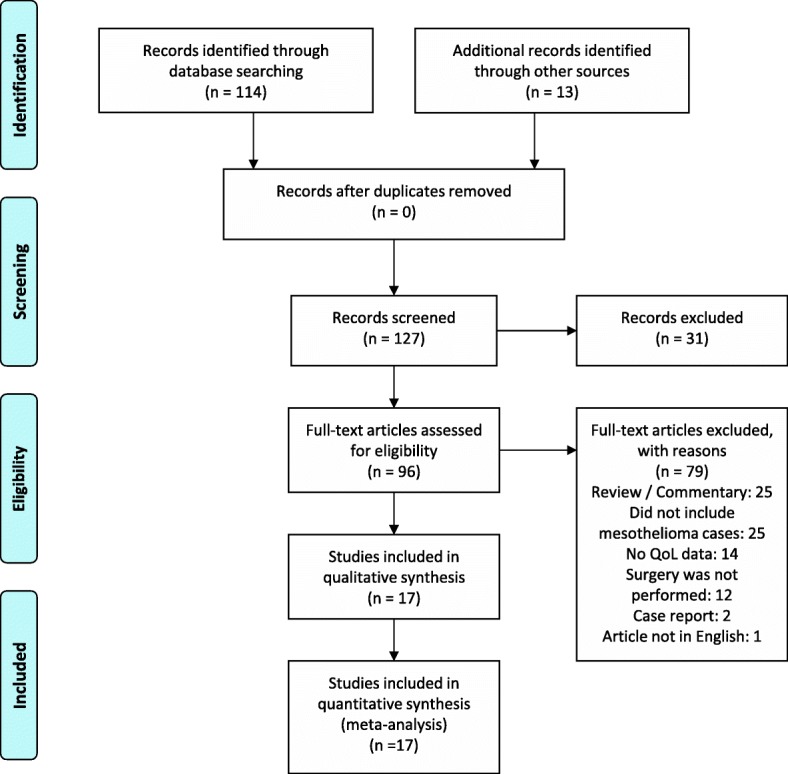
Preferred Reporting Items for Systematic Reviews and Meta-Analyses (PRISMA) flow diagram

The initial search yielded 96 potential publications. All abstracts were reviewed and 78 were excluded because the publication was a case report (*n* = 2), review/commentary (*n* = 25), did not include mesothelioma cases (*n* = 25) or quality of life data (*n* = 14), or reported on cases not treated with surgery (*n* = 12). The 18 articles remaining were reviewed in detail, and 1 was further excluded because the text was not in English [28]. This left 17 articles and 14 distinct datasets for a total of 659 pleural mesothelioma patients with QoL information (Fig. 1; Table 1). Of the 14 datasets, there were 2 RCTs, one comparing EPP to no surgery and the other comparing partial pleurectomy to talc pleurodesis. Data from these two RCTs were not comparing EPP and P/D, and only data from one arm of each RCT was used. The remaining data were from observational studies, both prospective (*n* = 10) and retrospective (*n* = 2). While most studies resulted in a good quality rating from the NIH’s quality assessment tool (Table 2), scrutiny of the included studies indicated the risk of bias. The majority of studies did not measure or account for the impact of confounders influencing surgical procedure choice and QoL outcomes. Additionally most studies did not provide a justification for the sample size used and the resulting sample sizes were small. All EPP studies had less than 50 patients per sample, while only 4/8 P/D articles had more than 50 cases, while the EPP vs P/D studies had less than 25 patients or 40 patients in each group (Table 1). These results should be interpreted in light of the risk of bias stemming from the observational studies included here.

**Table 1:** Description of the included studies for QoL after EPP or P/D for MPM

Author(s)	Study design	Years of data collection	Patient (N)	Case selection	Type of treatment	QoL measure	QoL measured at: baseline	3 months	6 months	1 year	> 2 years	Recurrence	QA^a^
EPP													
WEDER, RIBI [29, 30]	O (prospective; multi-center)	2000–2003	45	Clinical T1-T3, N0–2; M0, any histology	Neo-adjuvant chemo (pre-op; *n* = 45/61), EPP, radiotherapy (post-op; *n* = 36/45)	Rotterdam Symptoms checklist; SEIQoL	y (pre-op)	1, 3	y	y		Median 13.5 months	10/12
AMBROGI [31, 32]	O (prospective; single-center)	1997–2007	29	NR	EPP, adjuvant chemotherapy and radiotherapy (post-op)	SF 36, St George, symptoms, lung function, 6 min walk, cardiac EF, Karnovsky	y (pre-op)	y	y	y	2, 3	Median 19.5 months	8/12
ALVAREZ [33]	O (prospective; single-center)	2004–2007	16 (18 no EPP)	stage I, II, epithelioid, age < 70 years	EPP, chemotherapy, radiotherapy (post-op)	ECOG, Karnofsky	ECOG 0		y	y		18% at 6 months	8/12
TREASURE [34]	RCT (multi-center)	2005–2008	12 (19 no EPP)	T1–3, N0–1, M0, any histology	Chemotherapy (pre-op), EPP, Radiotherapy (post-op)	EORTC C30; LC 13	y (pre-op)	6 weeks, 3 months	6, 9	12, 18 months	y	75% at 6 months	NA
P/D													
BURKHOLDER [35] (MOLLBERG [36])	O (prospective; single- center)	2010–2011	36	Epithelioid, biphasic WHO PS 0–2	EPD (some talc, neoadjuvant chemotherapy (*n* = 3), adjuvant chemotherapy (*n* = 30)	EORTC C30, lung function	y (pre-op)	4	5, 7, 8			NA	9/12
SAUTER [37]	O (prospective; single-center	1988–1992	20	NR	Partial P (some chemotherapy, some radiation; post-op)	dyspnea, pain	y		y			Median 10 months	9/12
SOYSAL [38]	O (retrospective; single-center)	1974–1992	100	NR	56 P/D; 44 Partial P, chemotherapy (*n* = 24/100; post-op), radiotherapy (*n* = 31/100; post-op), chemo + radio (*n* = 20/100; post-op)	dyspnea, pain, cough		y	y			Median survival 17 months	4/10
MARTIN-UCAR [39]	O (prospective; single-center)	1997–2001	51	Exclude early stage	P/D palliative	symptoms	y (pre-op)	6 weeks, 3 months	y	y		53% at 6 months	9/12
BOLUKBAS [40]	O (prospective; single-center)	2010	16	NR	radical P, chemotherapy, radiation (post-op)	lung function	y (pre-op)	2 months				NA	9/12
RINTOUL [41]	RCT (multi-center)	2003–2012	73	NR	73 Partial P, 78 talc, some chemotherapy, radiotherapy (post-op)	EORTC C30, LC 13, Euro 5, lung function	y (pre-op)	1, 3	y	y		Median survival 13 months	NA
TANAKA [42]	O (prospective; single-center)	2013–2015	22	NR	P/D, some chemotherapy (pre-op)	SF-36, lung function	y (pre-op)					NA	8/11
VIGNESWARAN [43]	O (prospective; single-center)	2008–2015	114	NR	28 chemotherapy before P/D	EORTC C30	y (pre-op)	1	4–5, 7–8	10–11		Median survival 15.21 months	9/12
EPP VS P/D													
RENA [44]	O (prospective; single-center)	1998–2009	77	Stage I and II	40 EPP (*n* = 7 adjuvant chemotherapy; *n* = 33 neoadjuvant); 37 P/D (*n* = 6 adjuvant chemotherapy; *n* = 31 neoadjuvant)	EORTC C30	y (pre-op)		y		y	Median survival 14 and 11 months	8/12
PLOENES [45]	O (retrospective; single-center)	NR	48	NR	25 EPP; 23 P/D (intraoperative chemotherapy; *n* = 6)	lung function	y (pre-op)		y		y	Median survival 22 and 29 months	10/12
TOTAL CASES			659										

*ECOG* Eastern Cooperative Oncology Group Performance Status, *EORTC* European Organization for Research and Treatment of Cancer, *EPD* extended pleurectomy and decortication, *EPP* extrapleural pneumonectomy, *MPM* malignant pleural mesothelioma, *NA* not applicable, *NR* not reported, *O* observational, *P* pleurectomy, *P/D* pleurectomy decortication, *PS* performance status, *RCT* randomized controlled trial, *SEIQoL-DW* Schedule for the Evaluation of Quality of Life-Direct Weighting, *QA* Quality Assessment, *QoL* quality of life, *QA* was not applied to the two RCTs, *y* yes

^a^Number of yes/number of relevant questions from the NIH Quality Assessment Tool for Observational Cohort and Cross-Sectional Studies

**Table 2:** Results from The National Institutes of Health Quality Assessment Tool for Observational Cohort and Cross-Sectional Studies

Author(s)	1. Research Question	2. Study Population	3. Participation Rate	4. Population Criteria	5. Sample Size	6.Exposure	7.Time Frame	8.Exposure Varying	9. Exposure Measure	10. Exposure Count	11. Outcomes	12 Blind Outcomes	13. LTF	14. Confounders	Quality Assessment^a^
EPP															
WEDER, RIBI [29, 30]	Y	Y	Y	Y	Y	Y	Y	NA	Y	NA	Y	N	Y	N	10/12
AMBROGI [31, 32]	Y	Y	Y	Y	N	Y	Y	NA	Y	NA	Y	N	N	N	8/12
ALVAREZ [33]	Y	Y	N	Y	N	Y	Y	NA	Y	NA	Y	N	Y	N	8/12
P/D															
BURKHOLDER [35] (MOLLBERG [36])	Y	Y	Y	Y	N	Y	Y	NA	Y	NA	Y	N	Y	N	9/12
SAUTER [37]	Y	Y	Y	Y	N	Y	Y	NA	Y	NA	Y	N	Y	N	9/12
SOYSAL [38]	Y	Y	N	N	N	N	NR	NA	Y	NA	Y	N	NR	N	4/10
MARTIN-UCAR [39]	Y	Y	Y	Y	N	Y	Y	NA	Y	NA	Y	N	N	Y	9/12
BOLUKBAS [40]	Y	Y	Y	Y	N	Y	Y	NA	Y	NA	Y	N	Y	N	9/12
TANAKA [42]	Y	Y	Y	Y	N	Y	Y	NA	Y	NA	Y	N	NR	N	8/11
VIGNESWARAN [43]	Y	Y	Y	Y	N	Y	Y	NA	Y	NA	Y	N	N	Y	9/12
EPP VS P/D															
RENA [44]	Y	Y	Y	N	N	Y	Y	NA	Y	NA	Y	N	Y	N	8/12
PLOENES [45]	Y	Y	Y	Y	N	Y	Y	NA	Y	NA	Y	N	Y	Y	10/12

^a^Number of yes / number of relevant questions

*Y* yes, *LTF* lost to follow-up, *NA* not applicable, *NR* not reported

Full Question Key: 1. Was the research question or objective in this paper clearly stated? 2. Was the study population clearly specified and defined? 3. Was the participation rate of eligible persons at least 50%? 4. Were all the subjects selected or recruited from the same or similar populations (including the same time period)? Were inclusion and exclusion criteria for being in the study prespecified and applied uniformly to all participants? 5. Was a sample size justification, power description, or variance and effect estimates provided? 6. For the analyses in this paper, were the exposure(s) of interest measured prior to the outcome(s) being measured? 7. Was the timeframe sufficient so that one could reasonably expect to see an association between exposure and outcome if it existed? 8. For exposures that can vary in amount or level, did the study examine different levels of the exposure as related to the outcome (e.g., categories of exposure, or exposure measured as continuous variable)? 9. Were the exposure measures (independent variables) clearly defined, valid, reliable, and implemented consistently across all study participants? 10. Was the exposure(s) assessed more than once over time? 11. Were the outcome measures (dependent variables) clearly defined, valid, reliable, and implemented consistently across all study participants? 12. Were the outcome assessors blinded to the exposure status of participants? 13. Was loss to follow-up after baseline 20% or less? 14. Were key potential confounding variables measured and adjusted statistically for their impact on the relationship between exposure(s) and outcome(s)?

### QoL after EPP

There were four datasets including QoL after EPP, 3 observational and 1 from one arm of a RCT, for a total of 102 patients. The study by Weder [29] utilized the Rotterdam Symptom Checklist, a self-report measure to assess physical and psychological quality of life, in 45 patients treated with neo-adjuvant chemotherapy, EPP and then potential adjuvant radiation. QoL was assessed preoperatively and postoperatively at 1, 3, 6 and 12 months. Psychological distress returned to values similar to baseline at 6 months. Physical symptoms declined at 1 month (− 16.7) but improved 6 months following surgery (− 4.3). While QoL did not return to baseline after 6 months (− 8.3), tiredness, shortness of breath and chest pain were worse at 1 month, but returned to baseline values at 6 months [30]. These same patients were also administered the Schedule for the Evaluation of Quality of Life-Direct Weighting (SEIQoL-DW) which is an individually driven QoL measure in which the patient determines the five QoL domains that are most important to him/her and then rates those 5 domains. Overall QoL scores as measured by the SEIQoL-DW decreased immediately after surgery, returned to baseline values at 3 months, but then decreased from baseline values at 6 months [29].

Ambrogi [31, 32] evaluated 29 consecutive patients who underwent neoadjuvant chemotherapy, EPP and adjuvant radiation. An extensive list of QoL measurements were reported at baseline and following surgery for up to 3 years of follow-up. Although lung and cardiac function were stable at 6 months, these had significantly deteriorated at 12 months. Pain, dyspnea, cough and fever initially improved at 3 months following surgery, but deteriorated again at 12 months, as did the Karnofsky Index, a 100-point measure of performance status. The SF-36, a 36-item survey of physical and mental health summary scores, improved across all domains at 3 months, but only the physical QoL domains remained above baseline at 12 months, while at 24 months, both physical and mental health scores decreased from baseline. Similar results were obtained by the St. George respiratory questionnaire.

Two studies compared QoL after EPP to QoL after no surgery; one study was observational and the other was from one arm of an RCT. Alvarez [33] studied 16 patients with stage I or II epithelioid mesothelioma who were Eastern Cooperative Oncology Group (ECOG) 0, younger than 70 years of age, and were treated with EPP followed by chemo and radiotherapy. ECOG and the Karnofsky Index were measured at 6 months (ECOG: 1.0; Karnofsky: 74) and 1 year (ECOG: 0.8; Karnofsky: 82) after surgery, with no baseline measurements available. In comparison, patients who did not undergo surgery (*n* = 18) demonstrated a stable mean ECOG of 1.7 and a Karnofsky Index score of 46 at both 6 and 12 months. Treasure et al. [34] conducted a feasibility trial, randomizing patients to receive EPP or no surgery. In this study, 12 patients underwent induction chemotherapy, EPP and adjuvant radiation. QoL was measured with the EORTC C-30 survey and the EORTC lung cancer-specific QoL questionnaire (LC-13) and results were compared to the control group undergoing chemotherapy only. Median QoL scores were lower in the EPP group as compared to the no surgery group at all time points, but particularly at 6 weeks (33.3 versus 75, respectively). However, none of the group differences were statistically significant.

### QoL after P/D

There were 8 studies with a total 432 patients evaluating QoL after P/D. Burkholder [35, 36] reported on 36 P/D patients with QoL data from the EORTC C30 questionnaire at baseline and up to 8 months after surgery. In some patients, neoadjuvant chemotherapy was given and/or pleurodesis was also performed. Among patients with WHO PS 0, baseline QoL scores were significantly higher as compared to PS 1 patients. Among the PS 0 patients, no post-operative change was observed in global health or function and symptoms scores, except for emotional function, which improved significantly during follow-up. PS 1 and PS 2 patients demonstrated improvement at 4–5 months and further improvement at 7–8 months in all QoL and symptom domains. PS 0 patients demonstrated a significant decrease in all lung function parameters, whereas no change was observed in PS 1 and PS 2 patients. In another study, Sauter [37] included 36 patients treated with partial pleurectomy who underwent different combinations of chemotherapy and radiation. Symptoms were collected at baseline and during follow-up using the five grades (0–4) of pulmonary symptoms listed in the National Cancer Institute Common Toxicity Criteria. Dyspnea improved in 47% of patients after surgery, while pain only improved in 21%. However, no follow-up time frame was given in the article regarding the assessment of symptoms and pain.

Soysal [38] reported QoL symptoms at baseline and 6 month follow-up from 100 consecutive patients who underwent P/D or partial pleurectomy over a 19 year period. At 6 months, 71% of patients reported decreased chest pain, 40% had decreased cough, 37% had decreased dyspnea, and 30% reported decreased chest constriction. The study by Martin-Ucar [39] reported on symptoms after P/D in 51 consecutive malignant mesothelioma patients, utilizing the Medical Research Council Dyspnoea Score and assesing pleuritic chest pain on a four-point scale (not at all, a little, moderate and severe). Significant improvement in dyspnea and pain scores were observed at 6 weeks and 3 months. Bolukbas [40] included 16 patients treated with radical pleurectomy followed by chemotherapy and radiation. Lung function was measured at baseline and 2 months following treatment. All functional parameters improved from baseline to follow-up. Rintoul [41] completed an RCT comparing partial pleurectomy to talc pleurodesis in 151 patients from 2003 to 2012. EORTC C30, EuroQoL 5D and the Lung Cancer LC-13 questionnaires were utilized to assess QoL at baseline and up to 1 year after surgery. While EuroQoL data showed a significant decrease at 1 month, these scores retuned to baseline at 3 months and continued to improve at 12 months. The EORTC C30 physical, cognitive, and role functioning scales were lower than baseline values at 1 month after surgery, but then returned to preoperative levels at 3, 6 and 12 months. The emotional functioning and social functioning scales as well as global health scores also improved at 12 months compared to preoperative values. Forced expiratory volume in 1 s (FEV1) and forced vital capacity (FVC) immediately improved 1 month after surgery and persisted at 12 months.

Tanaka [42] studied 22 patients who underwent P/D and measured physical function (handgrip strength, knee extensor strength test, 6-min walk distance), pulmonary function (FVC, FEV1) and QoL through the SF-36. At follow-up, handgrip strength, 6-min walk distance, FVC, and FEV1 statistically significantly decreased, as well as physical functioning, body pain and vitality as measured by the SF-36.

Vigneswara [43], evaluated 114 patients, 28 of which had chemotherapy before P/D. QoL was assessed using the EORTC C30 at baseline and at 1, 4–5, 7–8, and 10–11 months following surgery. At baseline, patients with PS 1–2, non-epithelioid histology, and large pathological tumor volume had low QoL, but demonstrated improvement over the follow-up period. Symptoms of dyspnea and fatigue decreased significantly following surgery, while physical and emotional functioning improved over time. At 7–8 months, overall QoL, physical and social functioning, lack of appetite, pain and insomnia showed the most improvement.

**Fig. 2:**
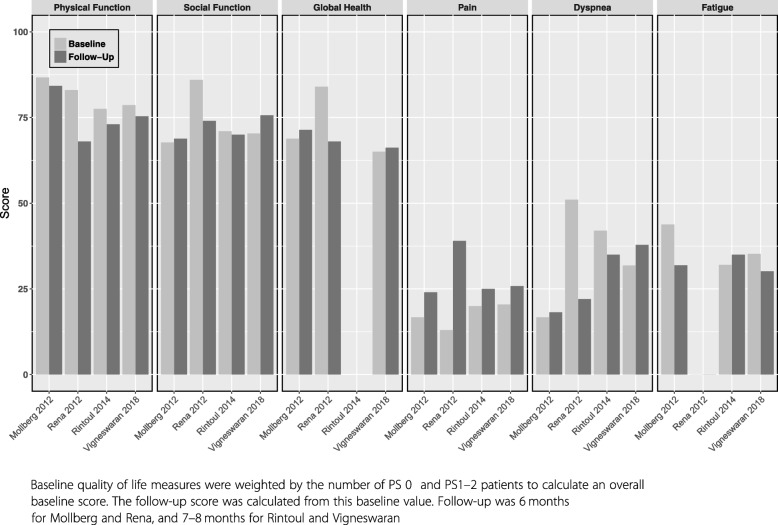
EORTC C30 quality of life and symptoms scores changes after P/D.

A comparison across studies that measured QoL components and symptoms before and after surgery for the studies that used the EORTC C30 questionnaire is reported in Fig. 2 [36, 41, 43, 44]. At 6 months, physical and social functioning and global health were not yet back to pre-surgery values, and pain was still the main symptom reported, while dyspnea and pain scores showed variability compared to pre-surgery.

**Fig. 3:**
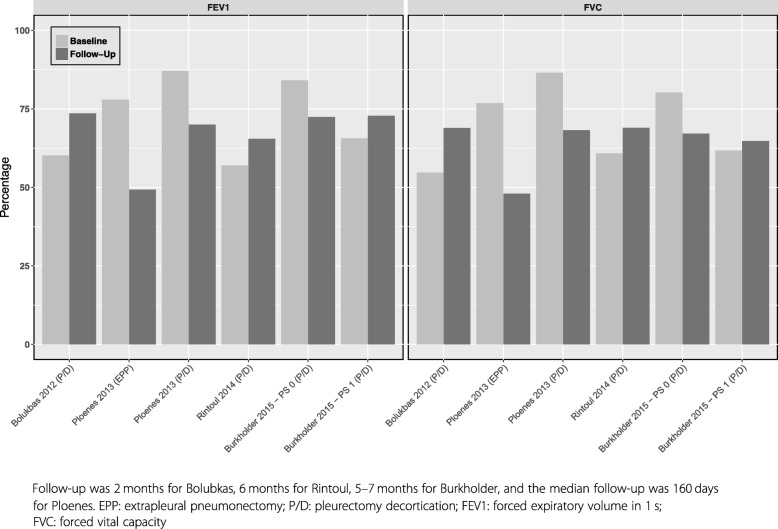
Lung Function after P/D and EPP.

Four studies reported lung function after P/D (Fig. 3); Bolukbas [40] and Burkholder [35] studied PS 1 patients and observed an improvement in FEV1 and FVC following P/D; this improvement was not present in PS 0 patients [35]. Rintoul [41] reported increased FEV1 (57 to 65.5%) and FVC (60.8 to 69.0%) 6 months post video-assisted thoracoscopic partial pleurectomy. Tanaka [42] reported statistically significant decreases in mean (± standard deviation) FVC1 (3.28 ± 0.85 Liters to 1.92 ± 0.4 Liters; *p* < 0.0001) and mean FEV1 (2.35 ± 0.59 Liters to 1.57 ± 0.37 Liters, *p* < 0.001) following P/D; the authors did not report FEV or FVC as a percent.

### QoL comparison between EPP and P/D

**Fig. 4:**
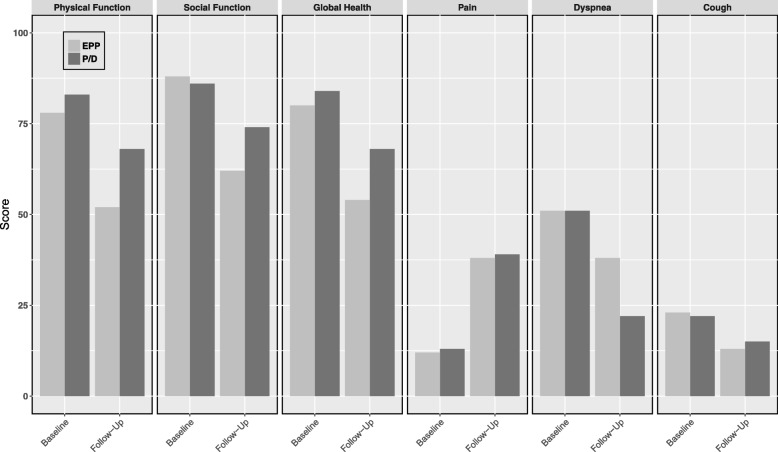
Comparison of baseline and follow-up (6 months) EORTC C30 quality of life and symptoms - P/D versus EPP (from Rena, 2012 [44]). EPP: extrapleural pneumonectomy; P/D: pleurectomy decortication

Two studies compared QoL following EPP and P/D. Ploenes et al. [45] analyzed lung function at baseline and at another time point between 1 and 29.7 months following surgery in 25 patients who underwent EPP and 23 who underwent P/D (Fig. 3). EPP patients had a significantly reduced pulmonary function compared to P/D patients. Rena et al. [44] studied 77 patients with stage I or II mesothelioma, 40 of whom underwent EPP and 37 underwent P/D. The EORTC C30 questionnaire was administered at baseline and at 6 and 12 months after surgery and both procedures resulted in significant impairment of all EORTC C30 variables at 6 months (Fig. 4). The severity of QoL impairment was worse in EPP patients and only P/D patients returned to baseline levels at 12 months.

## Discussion

This review of the current literature on QoL after surgical resection for MPM suggests that symptoms, lung function parameters, and physical and social functions remain compromised for 6 months following surgery. However, from the limited and low quality data available comparing the two surgical procedures, it would appear that P/D patients fared better than EPP patients across all QoL measures. This result likely reflects the increased morbidity of EPP compared to P/D. These conclusions, however, need to be viewed as preliminary given the lack of published RCTs and the heavy reliance on observational studies for this review.

As most studies in the literature are retrospective, it is possible that patients in each cohort were treated with the appropriate surgical procedure that considered their clinical characteristic and/or comorbidities, with these same factors potentially independently impacting QoL. This type of selection bias would be accounted for in a RCT comparing the two procedures. In one of the two studies comparing the two procedures in an observational retrospective design [45], lung function was comparable at baseline, but was significantly worse at 6 months among EPP patients as compared to P/D patients. In the other [44], P/D and EPP patients were very similar at baseline, but the physical, social functioning and global health scales were worse after EPP in comparison to P/D. Therefore, even when one considers how selection bias might affect the results of retrospective studies, the conclusions may still show some advantages for P/D. Comorbidities that impact choice of surgical resection for an MPM patient generally associate sicker patients with P/D (as they would not likely tolerate EPP) and any impact of these comorbidities on QoL should favor EPP. With regard to extent of disease, critics of P/D may suggest that patients undergoing EPP have higher tumor burden and this might impact QoL. However, most studies in the review stratified by stage or otherwise excluded patients with advanced disease. The reviewed literature clearly suggests that there is a delicate balance between maximum prolongation of survival and preservation of quality of life in mesothelioma surgical patients, and that the relative priorities of each patient should be considered when deciding on the extent of surgery. Patients should also be made aware that it is unrealistic to expect this extensive surgery to improve QoL, but rather just preserve the preoperative values.

This review highlights serval gaps and limitations in the existing literature and identifies the low quality of available evidence to compare QoL after P/D and EPP. The number of datasets that included QoL measures was relatively small (14 datasets) especially given the extensive literature on MPM surgery. There was only data from one arm of two RCTs, with the remaining data from observational studies. Despite high scores on the NIH Quality Assessment Tool (Table 2), low quality evidence exists to perform the QoL comparison, consistently stemming from low sample size, lack of randomization, and the presence of confounders. Each dataset involved a small number of patients, from 12 to a maximum of 114, which is a key limitation in data stemming from predominately observational study designs. There was also variability in the years of data collection and the length of the studies, which may make it difficult to compare the effects of surgical procedures that could have changed in technicality and invasiveness over time. Similarly this long time span introduces confounders which influence QoL and limit the ability to perform a comparison.

The instruments used to measure QoL were also highly variable and often non-comparable with each other, thus making it difficult to quantify the effect of each surgical approach on QoL. Another source of variability was that QoL measurement was often performed at baseline and then after surgery at different time points, from 1 to 6 months, and occasionally at 1 year. The use of different QoL measures at different times limited the amount of studies included in comparisons. Other treatments, such as chemotherapy and radiation, were often administered, but their effect on QoL was not accounted for in the publications. Additionally these adjuvant treatments varied by study, sample size, and administration (pre-op, post-op, both, neither), which accounts for variability in QoL determinations. Patients included in the QoL studies were very heterogeneous in age, stage, and comorbidities. While most radical resections are performed via thoracotomy, it is also possible that Video-Assisted Thoracoscopy (VATS) approaches were used, and while this may have had a differential impact on QoL, these details were missing in the publications. There were more QoL data on patients who underwent P/D than EPP, and it is possible that if more EPP patients were included, the results might differ. Furthermore, whenever QoL questionnaires are used, one must consider that the subset of patients who respond may exclude the most ill (and lowest QoL) patients, who are unable to respond, or those with better performance status who prefer to continue with their daily activities rather than remain involved in clinical studies.

Accounting for the net direction of these biases would allow for a more accurate quantification of change in QoL after MPM surgery. Statistical aggregation of the individual data was not performed due to the heterogeneity across studies and the lack of abundant and comparable data. Future studies on MPM treatment and outcomes should include QoL measurements acquired at baseline and multiple time intervals, stratified according to treatment, including multimodal therapies. Since no RCTs compared EPP and P/D, data was gathered from observational studies, or in two instances, one arm of an RCT. Further RCTs are needed to directly compare EPP and P/D; the ongoing MARS-2 trial may be able to add unbiased information on the role of P/D on QoL of mesothelioma patients [46]. QoL results are needed to inform patients and treating clinicians to guide treatment choices in MPM.

## Conclusions

This literature review shows that there is a limited amount of studies on QoL changes after P/D and EPP for MPM, and that these studies use different methodologies for inclusion criteria, QoL measures, and methods for reporting results. Although the existing evidence is limited and of low quality, it suggests that QoL is better for patients undergoing P/D compared to EPP for an extended period following surgery. QoL outcomes should be strongly considered when choosing type of surgery for MPM, and possible effects on lung function and QoL should be discussed with patients when presenting surgical treatment options.

## Abbreviations

ECOG: Eastern Cooperative Oncology Group
EORTC: European Organization for Research and Treatment of Cancer
EPP: Extrapleural pneumonectomy
FEV1: Forced expiratory volume in 1 second
FVC: Forced vital capacity
MPM: Malignant pleural mesothelioma
P/D: Pleurectomy decortication
PS: Performance Status
QoL: Quality of life
RCT: Randomized controlled trial
SEER: Surveillance, Epidemiology, and End Results
SEIQoL-DW: Schedule for the Evaluation of Quality of Life-Direct Weighting
VATS: Video-Assisted Thoracoscopy
